# Effectiveness of ultra-/very-high-frequency oscillations combined with helium–oxygen gas mixture in a rabbit model

**DOI:** 10.1038/s41598-024-77703-0

**Published:** 2024-10-29

**Authors:** Louis Akiyama, Shinobu Tatsunami, Mieko Akita, Naoki Shimizu

**Affiliations:** Department of Paediatrics, St. Marianna University School of Medicine, 2-16-1 Sugao Miyamae, Kawasaki, Kanagawa 216-8511 Japan

**Keywords:** High frequency oscillatory ventilation, Helium, Ultra-high frequency, CO_2_ excretion, Oscillation, Animal experiment, Biomedical engineering, Paediatric research, Respiratory tract diseases

## Abstract

High-frequency oscillatory ventilation (HFOV) at frequencies of approximately 15 Hz is associated with optimal CO_2_ excretion. Higher frequencies using a nitrogen–oxygen gas mixture worsen CO_2_ excretion. An in vitro experiment using HFOV and a helium–oxygen gas mixture showed a significant increase in CO_2_ transport, which increased with increases in ventilation frequency. We hypothesised that in HFOV, the change in the arterial partial pressure of CO_2_ (PaCO_2_) would be greater at frequencies above 15 Hz when combined with helium–oxygen gas mixture administration. We tested this hypothesis in a hypoventilated healthy rabbit model by administering a helium–oxygen gas mixture at 15, 25, 35, and 45 Hz frequencies. One-way repeated measures ANOVA showed a significant decrease in PaCO_2_ among the four ventilation frequency groups. Post-hoc analysis showed significant differences between 15 and 35 Hz frequencies and between 15 and 45 Hz frequencies. The mean (standard error) decrease of PaCO_2_ was 10.8 (2.2), 14.1 (2.3), 21.3 (3.3), and 23.1 (2.5) mmHg at 15, 25, 35, and 45 Hz, respectively. Combination therapy of helium–oxygen gas mixture and high-frequency oscillation using ultra/very high frequencies (35–45 Hz) was associated with a greater PaCO_2_ decrease than that using the standard frequency (15 Hz).

## Introduction

Helium–oxygen gas mixture has been reported to improve gas exchange during mechanical ventilation^1–6^. Helium–oxygen gas mixture as an adjunct therapy with conventional mechanical ventilation (CMV) has been used to treat exacerbations of chronic obstructive pulmonary disease in adults^5,6^, refractory status asthmaticus in children^7^, and congenital diaphragmatic hernia in neonates^8^. Moreover, helium–oxygen gas mixture has been used in combination with high-frequency oscillatory ventilation (HFOV), although improvement in respiratory status has only been documented in case reports, and this combination therapy is not currently an established treatment option^9^. We previously reported that using a helium–oxygen gas mixture in combination with HFOV significantly improved carbon dioxide (CO_2_) elimination in animal models compared to combination therapy with CMV^10^.

HFOV was first developed by Drs. Miyasaka, Bohn, and Bryan in Toronto^11,12^. HFOV delivers very small tidal volumes that are smaller than the anatomic dead space. Hence, the mechanism of CO_2_ elimination is considered to be molecular diffusion rather than classical ventilation^13–15^. Imai et al. reported the lung-protective effects of HFOV with decreased neutrophil infiltration in the alveoli and lower levels of inflammatory cytokines, such as tumour necrosis factor-α, in broncho-alveolar lavage fluid than those observed with CMV use^16^.

The optimal frequency for CO_2_ elimination was found to be approximately 15 Hz in a beagle dog model (mean weight, 11 kg)^11,12^. Since these initial investigations, HFOV has been used in several clinical settings at a frequency of 15 Hz or even lower. However, it is crucial to differentiate between the use of HFOV in adults and paediatric patients. Typically, because the ventilation frequency of HFOV should be close to the resonant frequency, frequencies around 5 Hz have been applied in adults, whereas higher frequencies, around 15 Hz, are used in paediatric and neonatal settings^8,17,18^. A study using a rabbit model (approximately 3 kg body weight, similar to newborn infants) comparing the application of 15 Hz and 5 Hz frequencies showed no difference in CO_2_ elimination; however, histopathological changes, such as small airway injury and alveolar neutrophilic infiltration, were observed with a lower frequency of 5 Hz^19^. Thus, HFOV at higher frequencies may provide lung-protective effects, and the use of frequencies even higher than those previously used may have additional benefits as lung-protective ventilation. Consequently, there is a need to explore the potential of using higher frequencies for enhanced lung protection.

Currently, there is a lack of studies on HFOV using a higher frequency of 30 Hz or above. The poor CO_2_ elimination observed in early studies using ventilation with oxygen-enriched air at frequencies above 15 Hz may be attributable to the use of a nitrogen–oxygen gas mixture without helium^11,12^. In animal experiments or clinical trials investigating HFOV used in combination with a helium–oxygen gas mixture (rather than a nitrogen–oxygen gas mixture), the HFOV has been used at a frequency of 15 Hz or lower^9,20–24^. However, there is a lack of data showing the optimal frequency for CO_2_ elimination or the effect of using higher frequencies on CO_2_ elimination with a helium–oxygen gas mixture.

Helium is an inert, non-toxic gas and has non-lasting effects on the human body^1^. Since the density of a helium–oxygen gas mixture is approximately one-third of that of a nitrogen–oxygen gas mixture (oxygen 21%), the Reynolds number is decreased. The Reynolds number is a dimensionless quantity used to determine whether a flow is smooth or turbulent. It is directly proportional to density, velocity, and length and inversely proportional to viscosity. Therefore, administering a helium–oxygen gas mixture will convert a turbulent flow to a laminar flow, if the Reynolds number is sufficiently low^3–6^. As airway resistance is lower in laminar flow than that in turbulent flow, the conversion of turbulent flow to laminar flow results in lower airway resistance throughout the entire airway. Applying Poiseuille’s theory, airway resistance is inversely proportional to the fourth power of the radius of the trachea; accordingly, children and neonates have relatively high airway resistance since their airways are very narrow, and smaller endotracheal tubes tend to be used^3^. Therefore, the benefits of reduced airway resistance can be more efficiently achieved. In addition, since the density of a helium–oxygen gas mixture is lower than that of a nitrogen–oxygen gas mixture, according to the law of constant mean kinetic energy of gases, which states that when the average kinetic energy is constant, gases with smaller molecular weights require increased velocity, the velocity of the helium–oxygen gas mixture is increased. This increased velocity of the gas enhances the rate of flow through the trachea, which is beneficial in reducing respiratory workload. Based on these properties of helium, airway resistance and work of breathing will be reduced when the helium–oxygen gas mixture is administered during both CMV and HFOV^5–9,25^. Moreover, considering that helium has a higher diffusion coefficient of CO_2_ than oxygen or nitrogen, the administration of helium–oxygen gas mixture would be expected to promote CO_2_ excretion^3,9,20,26^. Notably, a bench study showed that CO_2_ transport increased with increasing ventilation frequency, and the use of a helium–oxygen gas mixture as a carrier gas significantly augmented CO_2_ transport^27^.

We hypothesised that the effect of using HFOV with helium–oxygen gas mixture on CO_2_ elimination might increase with a ventilation frequency over 15 Hz, based on the physical properties of helium described above. Demonstrating this phenomenon would allow us to propose an innovative ventilation method based on the combination of a helium–oxygen gas mixture and ultra-/very-high-frequency oscillation (U/VHFO), with a higher frequency than that used in conventional HFOV. This study aimed to test this hypothesis in an animal model.

## Methods

### Ethical statement

This study was approved by the Institutional Animal Care and Use Committee of St. Marianna University School of Medicine, in Kanagawa, Japan (Approval No. 2110010, 2210006). This committee reviews research plans based on the "Guidelines for Proper Conduct of Animal Experiments" published by the Science Council of Japan ^28^. These guidelines call for following the 3R Principle (Replace, Reduce, Refine) in animal experiments, similar to the Guide for the Care and Use of Laboratory Animals (Institute for Laboratory Animal Research [ILAR]) in the United States. This study was conducted in accordance with the relevant guidelines and regulations, including the American Veterinary Medical Association (AVMA) guidelines for the anaesthesia and euthanasia of animals^29^, and is also reported according to the Animal Research Reporting In Vivo Experiments (ARRIVE) guidelines.

### Aim, design, and setting

The study using a rabbit model was performed at the animal experimental laboratory of the Department of Paediatrics, St. Marianna University School of Medicine. In this study, a 15 Hz frequency was used for traditional HFOV, and frequencies of 25, 35, and 45 Hz were used for U/VHFO. The primary endpoint was the change (decrease) in the arterial partial pressure of CO_2_ (PaCO_2_) by administration of a helium–oxygen gas mixture. The secondary endpoints were the changes (increases) in the arterial partial pressure of oxygen (PaO_2_), heart rate, and blood pressure following helium–oxygen gas mixture administration.

### Animal preparation

Animal preparation was performed following established procedures^10^. Six Japanese white rabbits (specific pathogen-free, mean body weight 3.04 kg, standard error 0.08 kg) were used for the study. Animals were anaesthetised by intramuscular administration of an induction anaesthetic cocktail containing medetomidine acid (0.17 mg/kg/dose), midazolam (1 mg/kg/dose), and butorphanol tartrate (1.7 mg/kg/dose). Animals were placed in the supine position under a radiant warmer for infants (V 3200®, Atom Medical Corporation, Tokyo, Japan). An additional warmer (Heater Mat for Small Animals, Type III KN-475–3-40®, Natsume Seisakusho, Tokyo, Japan) was inserted between the animal’s back and the bed of the radiant warmer to maintain a rectal temperature in the range of 39–40 °C during the experiment. Electrocardiogram (ECG), transcutaneous oxygen saturation (SpO_2_), and rectal temperature (Trec) were monitored using appropriate probes.

A peripheral venous line was placed in the posterior auricular vein using a 24G peripheral venous catheter (Jelco®, Smiths Medical Japan Ltd, Tokyo, Japan) and used to administer intravenous fluids and continuous intravenous anaesthesia and analgesia. An infusion of maintenance fluid (Veen D Inj®; Fuso Pharmaceutical Industries, Ltd., Osaka, Japan) was started through an intravenous line. Maintenance infusion was administered at a rate of 10 mL/kg/h for the first hour and then adjusted to a rate of 4 mL/kg/h. An arterial pressure line was placed in the posterior auricular artery using a 24G peripheral venous catheter (Jelco®). This arterial pressure line was used to collect blood for arterial blood gas (ABG) analysis and arterial blood pressure (ABP) monitoring.

The cervical intermuscular space was separated. Topical anaesthesia with 1% lidocaine (0.2 mL) was administered to the anterior surface of the trachea just before the incision of the tracheal wall. A non-cuffed endotracheal tube (inner diameter, 3.0 mm; Portex®, Smiths Medical Japan, Ltd., Tokyo, Japan) was inserted into the site of the tracheostomy, adjusted so that the tip of the endotracheal tube was 3 cm deep below the inferior end of the cricoid cartilage, and ligated and fixed to the muscles around the trachea using silk thread.

After confirming that the animal was ventilated in both lungs using a self-inflating ventilation bag, a 1 mg/kg dose of rocuronium was administered. Subsequently, a maintenance anaesthesia cocktail of medetomidine acid (0.1 mg/kg/h), midazolam (0.16 mg/kg/h), and rocuronium bromide (1 mg/kg/h) was initiated. The ventilator (Humming Vue®) was connected and was started in HFO mode. Continuous monitoring of ECG, SpO_2_, Trec, and ABP was performed using a biological information monitor (Intellivue MP30®; Philips, Amsterdam, The Netherlands). After all experiments had been performed, animals were euthanised with sodium secobarbital (100 mg/kg/dose).

### Helium and ventilator preparation

Two different types of gas mixtures were prepared for this experiment. One was helium–oxygen gas mixture, and the other was nitrogen–oxygen gas mixture. The helium–oxygen gas mixture was provided by connecting a 750 L gas cylinder containing helium (79%) and oxygen (21%) mixtures (Heliox®; Air Water Inc., Osaka, Japan) and a 750 L gas cylinder containing pure oxygen to a helium–oxygen blender (OA2015FVH®; Sanyu Technology Co., Ltd., Tokyo, Japan). The helium concentration (0–79%, O_2_ concentration 100–21%) and gas flow rate (0–10 L/min) were prescribed by the helium–oxygen blender. The nitrogen–oxygen gas mixture was provided by connecting a 750 L cylinder containing pure oxygen and a 750 L cylinder containing air (N_2_ 79%, O_2_ concentration 21%) to an air-oxygen blender (OA2015FV®; Sanyu Technology Co., Ltd., Tokyo, Japan). Nitrogen concentration (0–79%, O_2_ concentration 100–21%) and gas flow rate (0–10 L/min) were prescribed by the air-oxygen blender. While the helium preparation differed from those described in our previous experiments, preparation of the ventilator and respiratory circuit were conducted as previously described^10^.

The green tube was connected to the gas outlet of the helium–oxygen blender and the gas outlet of the air-oxygen blender, individually, and connected to the inlet of the Jackson–Rees system circuit using a 3-way valve to facilitate the flow of one of the gas mixtures. Therefore, the gas type is selected at the 3-way stopcock. The fraction of inspiratory oxygen (F_I_O_2_) is selected with the gas blender, and the residual intake gas is either helium or nitrogen. The flow rate of the gas mixtures was fixed at 8 L/min. The Jackson–Rees system bag was connected to the ventilator circuit. A Humming Vue® ventilator (Metran Co., Ltd.) prototype was used in the experiment. It is identical to the commercially available Humming Vue® except for its capability to increase the frequency to up to 50 Hz, which was modified by Metran Co., Ltd. This company confirmed that even at increased frequencies, the prototype operates according to the set stroke volume (SV).

The Humming Vue® ventilator is piston-driven, with oscillations generated by the piston’s movement. As the piston moves, it creates oscillations in the gas, and the SV is the volume of gas pushed out by the piston. This SV is measured by the volume principle. The direct movement of the piston ensures that the set SV is accurately maintained across various frequencies, and it works independently on gas mixture density. When operating the commercially available Humming Vue® in HFOV mode, the adjustable settings include inspiratory oxygen concentration, SV, frequency, mean airway pressure (MAP), base flow, and sigh pressure. In this particular HFOV model, the pressure amplitude (displayed as amplitude in the Humming Vue®) is not an adjustable parameter but a dependent factor, influenced by SV, frequency settings, and other related variables. Although the SV can be defined, the amplitude, which is measured at the distal end of the ventilator circuit, cannot be adjusted. Sigh pressure must be manually applied, as it does not activate automatically. Additionally, due to the piston’s direct movement, there are no settings for inspiratory time or inspiratory-to-expiratory time ratio, which is fixed at 1:1.

With the current modifications to the Humming Vue®, the frequency can be significantly increased. Although a port for amplitude measurements existed, amplitudes were not measured for two reasons: the ventilator circuit was filled with a helium–oxygen gas mixture, which may render measurements inaccurate, and the frequency exceeded the 17 Hz that can be set on the commercially available Humming Vue®, with no standardized method for measuring amplitude established within this ventilator.

**Fig. 1:**
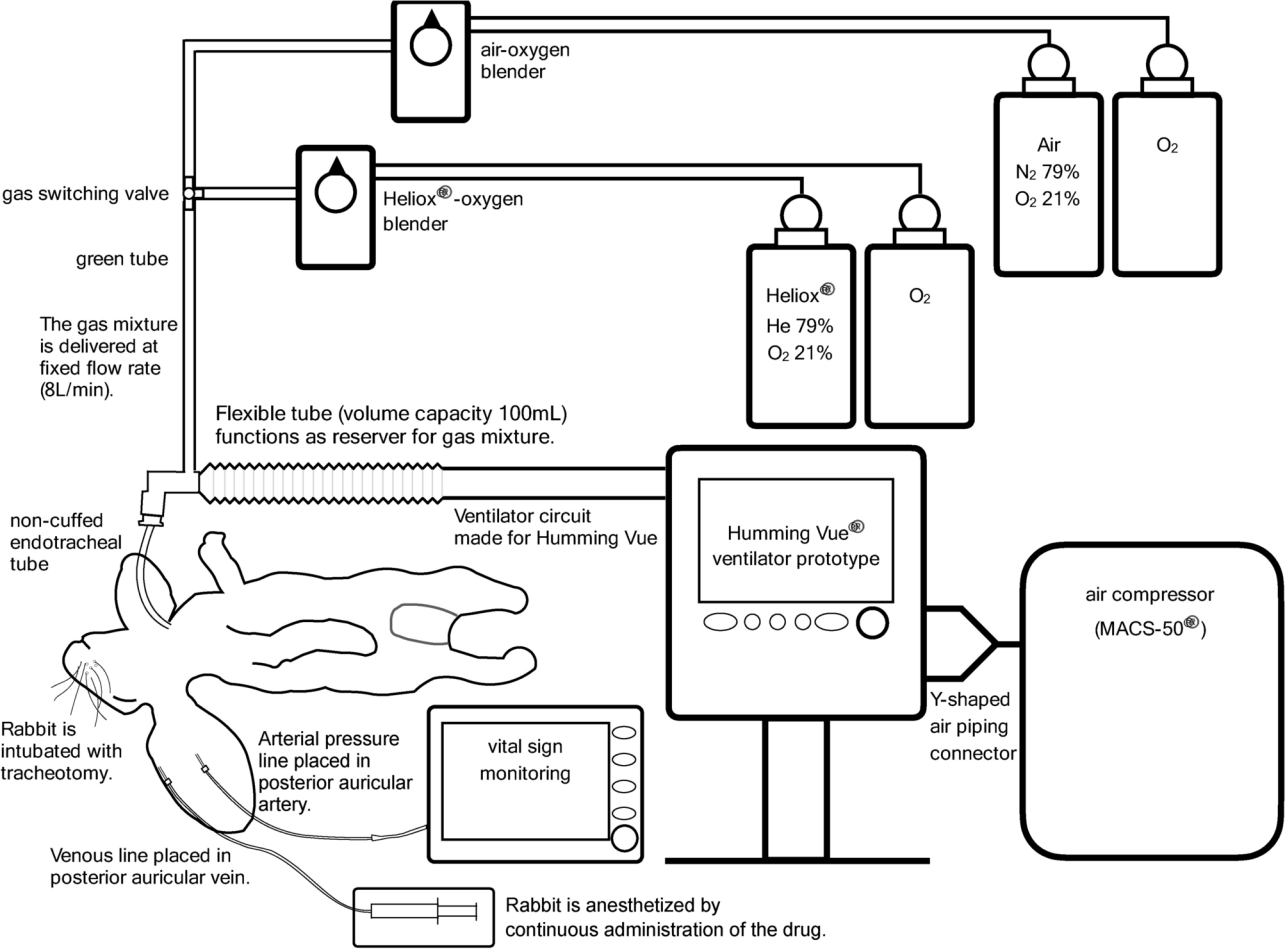
Schema of the experiment. Two different types of gas mixtures were prepared for this experiment. One was a helium–oxygen gas mixture, and the other was a nitrogen–oxygen gas mixture. The flow rate of the gas mixtures was fixed at 8 L/min.

An air compressor (MACS-50®; Tokibo International Co., Ltd., Osaka, Japan) was used as the ventilator’s driving source with a Y-shaped air piping connection made specifically for this experiment. A schematic diagram of the experimental system is shown in Fig. 1.

### Interventions and measurements

Animals were initially controlled in HFOV mode with F_I_O_2_, 1.0; MAP, 10 mmHg; SV, 10 mL; and frequency, 15 Hz. Sustained inflation of the HFO was performed repeatedly to recruit the collapsed lung, and ABG was sampled to confirm that PaO_2_ was above 400 mmHg in the condition of F_I_O_2_ 1.0. F_I_O_2_ 1.0 was used during the stabilisation phase of the animal model to prevent hypoxemia and other respiratory complications, thereby reducing the risk of unexpected animal deaths. After sustained inflation and confirmation that the PaO_2_ was above 400, we changed the ventilator settings as follows: F_I_O_2_, 0.3; fraction of inspiratory helium (F_I_He), 0; MAP, 10 mmHg; and SV, 10 mL. Ventilation frequency was randomly selected from 15 Hz, 25 Hz, 35 Hz, and 45 Hz. The initial adjustment of the ventilator settings aimed to maintain PaCO_2_ within the high range of 70–95 mmHg to simulate a hypoventilated state.

ABG was sampled 15 min after adjusting the ventilator settings. The target PaCO_2_ range was 70–95 mmHg, with the SV initially unchanged. If PaCO_2_ exceeded 95 mmHg, the SV was increased by 1 mL to bring PaCO_2_ back into the target range. Conversely, if PaCO_2_ fell below 70 mmHg, the SV was decreased by 1 mL to achieve the same target range. ABG was rechecked 15 min after these adjustments to confirm that PaCO_2_ was within the target range. The last ABG results collected during this adjustment phase were stored as ABG (before administration).

After this initial adjustment, the helium–oxygen gas mixture was started without changing the F_I_O_2_ (F_I_O_2_, 0.3; F_I_He, 0.7). The same HFO settings were maintained for 15 min, and ABG (during administration) was measured. Then, the administration of the helium–oxygen gas mixture was discontinued (F_I_O_2_, 0.3; F_I_He, 0). Using the same HFO settings for 15 min, a repeat ABG (after discontinuation) was measured. Each time the HFO setting or the inhaled gas mixture was changed, a sigh pressure of 20 mmHg was applied for 10 s twice. We defined the three phases of before administration, during administration, and after discontinuation as one cycle. Soon after completing one cycle at the initially selected frequency, we changed only the ventilation frequency to a different setting and started the steps of the before-administration phase of the next cycle, including checking the ABG results and adjusting the ventilator SV if necessary. Ultimately, we completed one cycle at each of the frequencies 15, 25, 35, and 45 Hz.

The ABG sample, obtained from an arterial line using a heparin-coated syringe, was analysed using the i-STAT 1 Analyzer® (Abbott, Chicago, Illinois, USA), and all ABG samples in this animal experiment were processed similarly.

### Statistical analyses

All data are presented as mean (standard error). Before the main analysis, we summarised the descriptive statistics of the PaCO_2_ and PaO_2_ data measured before the administration of helium–oxygen gas mixture. The association of PaCO_2_ and PaO_2_ data with frequency change was evaluated by Spearman’s rank correlation coefficient ρ.

To investigate the primary endpoint, we focused on the change in PaCO_2_ (ΔPaCO_2_), defined as the PaCO_2_ value during the administration of helium–oxygen gas mixture minus the PaCO_2_ value before the administration of helium–oxygen gas mixture. Then, ΔPaCO_2_ was analysed using one-way repeated measures analysis of variance (ANOVA) in the four frequency groups (from 15 to 45 Hz). In cases yielding a significant result with ANOVA, multiple pairwise comparisons between the 15 Hz group and the remaining frequency groups by means of paired t-tests were followed as post hoc tests. The *P*-value obtained in each pairwise comparison was adjusted by multiplying by the constant^30^ corresponding to the correction of the critical probability level using the Holm method^31^. The adjusted *P*-values were then compared to a critical probability such as 0.05, to avoid the inconvenience of presenting various critical probability values in pairwise comparisons.

Regarding the secondary endpoints, the change in PaO_2_ (ΔPaO_2_), defined as the PaO_2_ value during the administration of helium–oxygen gas mixture minus the PaO_2_ value before the administration of helium–oxygen gas mixture, was analysed following the same statistical methodology described above for ΔPaCO_2_. Additionally, Δ heart rate, defined as the heart rate during the administration of helium–oxygen gas mixture minus the heart rate before the administration of helium–oxygen gas mixture, and Δ blood pressure, defined as the blood pressure during the administration of helium–oxygen gas mixture minus the blood pressure before the administration of helium–oxygen gas mixture, were analysed using one-way repeated measures ANOVA in the four frequency groups. In cases where ANOVA yielded a significant result, multiple pairwise comparisons between the 15 Hz group and the remaining groups were conducted as post hoc tests using paired t-tests. All reported *P*-values except one-way repeated measures ANOVA are two-tailed, and *P*-values of less than 0.05 were considered statistically significant. All statistical analyses were performed using JMP® 15.2.0 (SAS Institute Inc., Cary, NC, USA). Statistical significance was defined as *P* < 0.05.

For the sample size estimation, we input a mean difference of 10.0, a standard deviation of 5.0, a significance level α of 0.05/3, and a power of 0.8 into the sample size estimation program of JMP® 15.2.0 for paired t-tests. We set the difference in PaCO_2_ to 10 mmHg as it was considered clinically significant and sufficient for an effect. The term 0.05/3 means the correction for multiple pairwise comparisons, with three comparisons between 15 Hz and other frequencies. The resulting minimum sample size was six animals. We adopted this sample size because it was similar to or slightly larger than the number of animals used in previous studies. In these previous studies, Japanese white rabbits, piston-generated HFOs similar to the one we used, and helium–oxygen gas mixture as the inhaled gas showed a significant decrease in PaCO_2_ with a sample size of 3–5 animals^23,24^. Additionally, we aimed to respect the 3R Principle in animal experiments by using as few animals as possible.

## Results

In this study, six Japanese white rabbits were prepared according to the experimental plan. Each rabbit underwent one cycle of interventions at four different frequencies: 15 Hz, 25 Hz, 35 Hz, and 45 Hz. None of the rabbits died during the experiment, and we successfully collected one cycle of data for each frequency from all rabbits. Consequently, we obtained a total of six cycles of data for each frequency using the six rabbits.

The ventilator settings, except for SV, were consistently maintained as follows for all frequencies before the administration of the helium–oxygen gas mixture: F_I_O_2_, 0.3; base flow, 10 L/min; and MAP, 10 cmH_2_O. As stated in the Methods section, no changes were made to these settings during the process. However, slight adjustments to the SV settings were necessary to maintain PaCO_2_ within the range of 70–95 mmHg before the administration of the helium–oxygen gas mixture. In accordance with the procedures detailed in the Methods section, the mean SVs (standard errors) for each frequency were as follows: 10.2 (0.2) mL at 15 Hz, 10.0 (0.0) mL at 25 Hz, 10.2 (0.2) mL at 35 Hz, and 10.3 (0.3) mL at 45 Hz.

For a comprehensive overview, we present the mean values and standard errors of PaCO_2_ and PaO_2_ obtained from ABG analysis before administration, during administration, and after discontinuation of the helium–oxygen gas mixture in Table 1. ABG analysis conducted before the administration of the helium–oxygen gas mixture revealed that as the frequency increased, PaCO_2_ tended to increase, while PaO_2_ tended to decrease (ρ-values regarding PaCO_2_ and PaO_2_ were 0.765 [*P* < 0.001] and − 0.787 [*P* < 0.0001], respectively).

**Table 1:** Comprehensive overview of PaCO_2_ and PaO_2_ values in arterial blood gas analyses before administration, during administration, and after discontinuation of helium–oxygen gas mixture.

Frequency	PaCO_2_ (mmHg)			PaO_2_ (mmHg)		
	Before administration	During administration	After discontinuation	Before administration	During administration	After discontinuation
15 Hz	74.9 (2.8)	64.1 (6.3)	73.4 (2.3)	79.2 (3.6)	89.0 (6.4)	81.7 (3.3)
25 Hz	79.6 (1.6)	65.5 (1.1)	81.6 (1,0)	76.8 (3.1)	91.3 (3.9)	74.7 (3.2)
35 Hz	82.7 (2.8)	61.4 (1.9)	80.1 (1.8)	67.8 (2.6)	94.7 (3.2)	70.5 (2.4)
45 Hz	90.0 (1.5)	66.9 (2.7)	89.8 (2.0)	52.8 (4.0)	85.7 (4.1)	53.7 (4.5)

Values are shown as mean (standard error).

PaCO_2_: arterial partial pressure of carbon dioxide, PaO_2_: arterial partial pressure of oxygen.

**Fig. 2:**
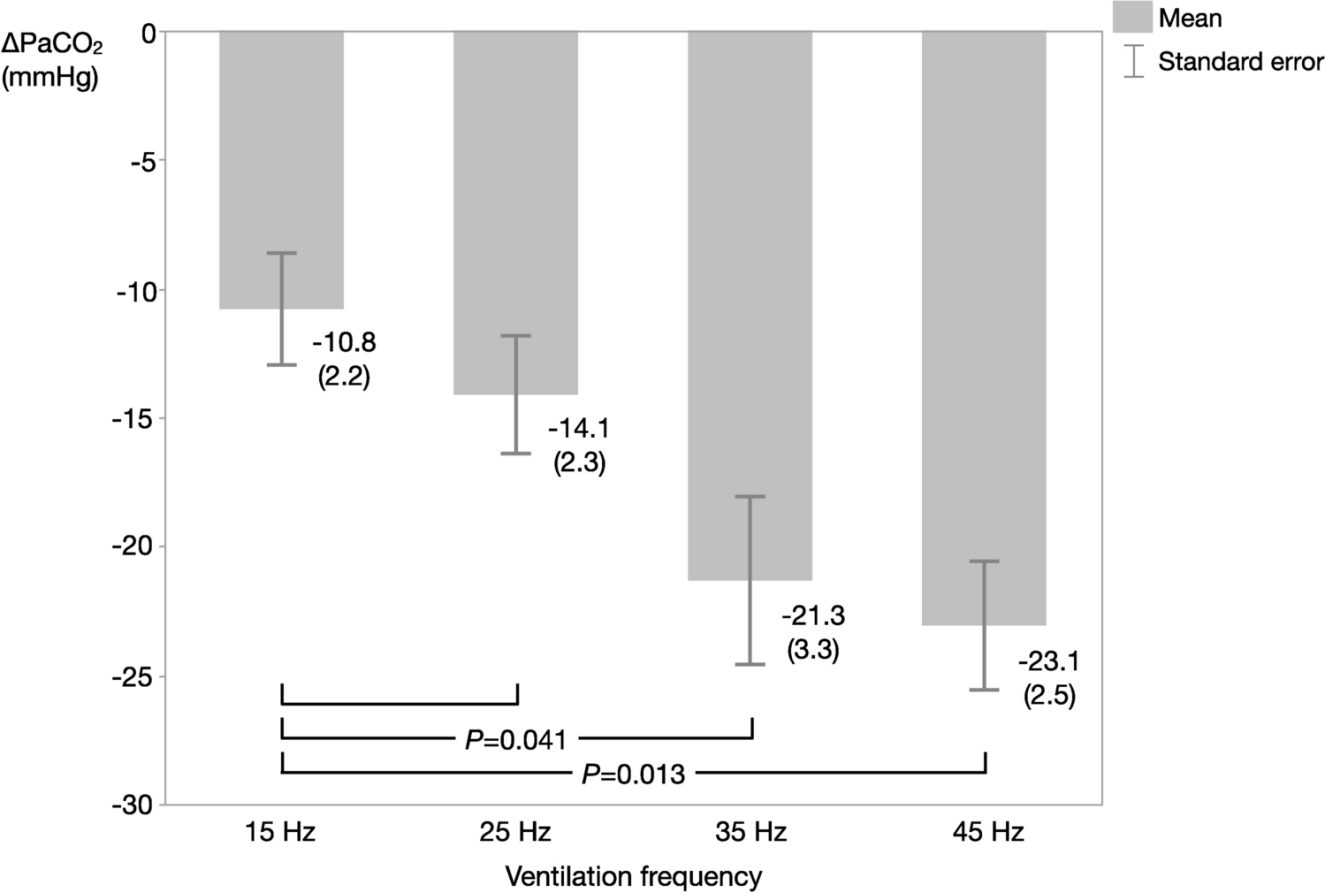
ΔPaCO_2_ at each ventilation frequency. One-way repeated measures ANOVA revealed a significant decrease in PaCO_2_ among the four ventilation frequency groups (*P* = 0.014). Post hoc analysis showed significant differences between 15 and 35 Hz frequencies and between 15 and 45 Hz frequencies. Values are shown as mean (standard error). The *P*-value for the post-hoc analysis was adjusted using the Holm method. ΔPaCO_2_: PaCO_2_ during administration of helium–oxygen gas mixture minus PaCO_2_ before administration of helium–oxygen gas mixture.

In subsequent analyses, we focused on ΔPaCO_2_ changes as the primary endpoint. The mean (standard error) ΔPaCO_2_ was -10.8 (2.2), -14.1 (2.3), -21.3 (3.3), and -23.1 (2.5) mmHg at 15, 25, 35, and 45 Hz, respectively. One-way repeated measures ANOVA revealed a significant ΔPaCO_2_ among the four ventilation frequency groups (*P* = 0.014). Post-hoc analysis using the Holm correction to adjust the *P*-value ^31^ revealed significant differences in ΔPaCO_2_ between 15 and 35 Hz frequencies and between 15 and 45 Hz frequencies (Fig. 2). There was no significant difference in ΔPaCO_2_ between 15 and 25 Hz frequencies.

**Fig. 3:**
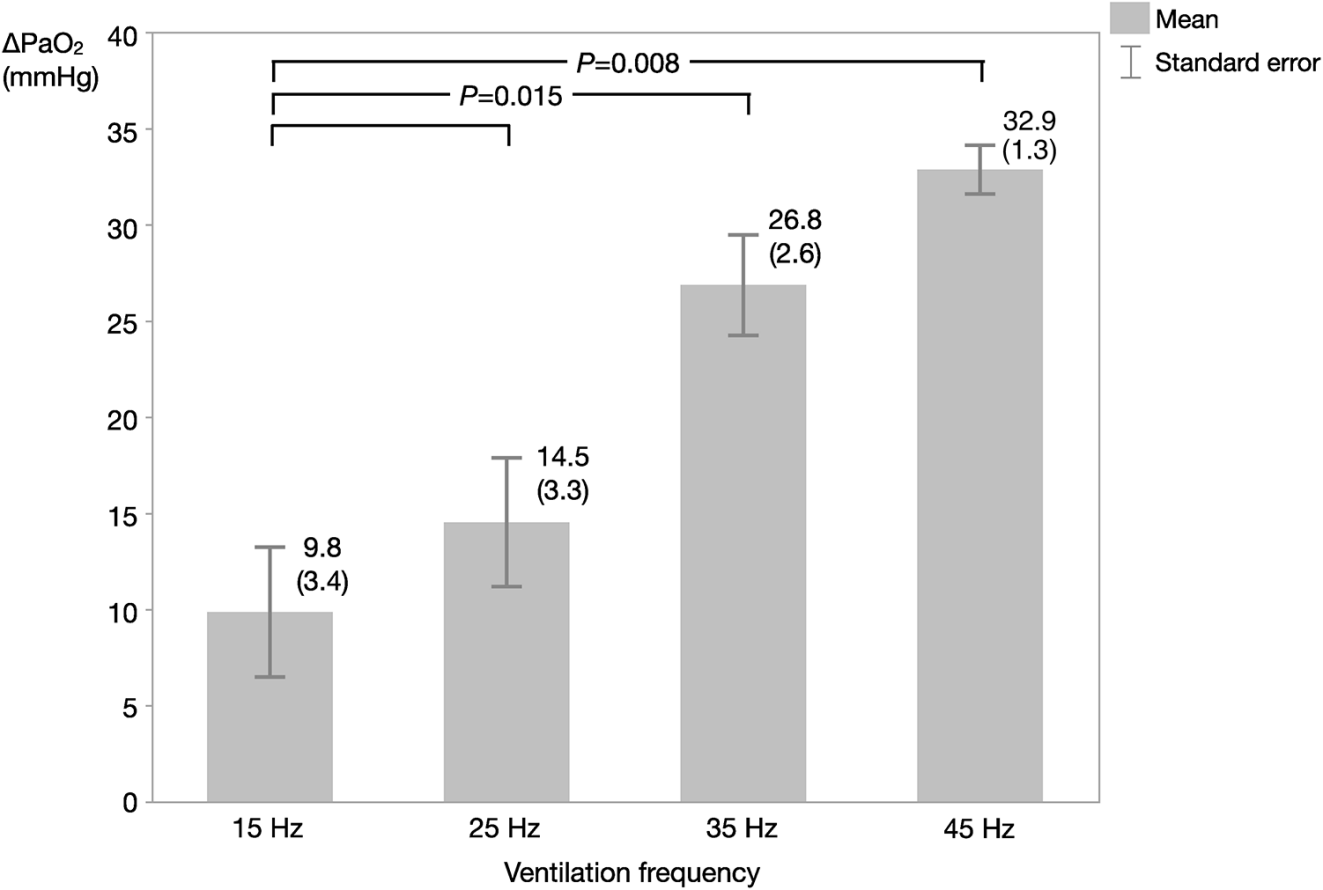
ΔPaO_2_ at each ventilation frequency. One-way repeated measures ANOVA showed a significant increase in PaO_2_ among the four ventilation frequency groups (*P* < 0.0001). The post hoc test showed significant differences in PaO_2_ between 15 and 35 Hz frequencies and between 15 and 45 Hz frequencies. Values are shown as mean (standard error). The *P*-value for the post-hoc analysis was adjusted using the Holm method. ΔPaO_2_: PaO_2_ during administration of helium–oxygen gas mixture minus PaO_2_ before administration of helium–oxygen gas mixture.

In addition to the primary endpoint, the secondary endpoints were analysed as follows. Regarding the change in PaO_2_ (ΔPaO_2_), one-way repeated measures ANOVA showed significant differences among the four ventilation frequency groups (*P* < 0.0001), and a successive post hoc test revealed that there were significant differences in ΔPaO_2_ between 15 and 35 Hz frequencies and between 15 and 45 Hz frequencies, with no significant difference between 15 and 25 Hz frequencies (Fig. 3).

The mean heart rate and blood pressure values at the time of blood sample collection for ABG analysis are shown in Table 2. One-way repeated measures ANOVA did not show any significant difference in both Δ heart rate (*P* = 0.0504) and Δ blood pressure (*P* = 0.1602).

**Table 2:** Heart rate and mean blood pressure at the time of arterial blood gas analysis.

Frequency	Heart rate (beats/minute)			Mean blood pressure (mmHg)		
	Before administration	During administration	After discontinuation	Before administration	During administration	After discontinuation
15 Hz	157.2 (9.5)	142.8 (6.6)	152.2 (7.1)	68.0 (3.3)	69.4 (3.3)	65.9 (3.5)
25 Hz	155.3 (7.6)	138.8 (4.5)	156.8 (7.0)	66.8 (3.6)	69.2 (4.4)	62.3 (3.3)
35 Hz	160.3 (6.8)	126.2 (5.0)	149.0 (8.0)	63.8 (3.3)	70.2 (3.0)	64.9 (2.2)
45 Hz	155.5 (6.9)	136.5 (6.7)	149.3 (8.2)	66.2 (3.6)	70.7 (3.1)	64.5 (4.1)

Values are shown as mean (standard error).

## Discussion

This study revealed that CO_2_ elimination by U/VHFO with helium–oxygen gas mixture increased as the ventilation frequency increased from 15 to 45 Hz. This result suggested that the optimal frequency of U/VHFO may be higher than 15 Hz in the combination therapy with the helium–oxygen gas mixture. These findings support further investigations into the efficacy of combination therapy using U/VHFO and helium–oxygen gas mixture for the treatment of hypercapnic respiratory failure. This study demonstrated that U/VHFO with higher frequency and/or helium–oxygen gas mixture administration did not cause any circulatory side effects, such as tachycardia or hypotension.

Regarding PaCO_2_, before the administration of the helium–oxygen gas mixture, PaCO_2_ increased with ventilation frequency. This result was similar to that of a previous study, in which optimal CO_2_ excretion was found at 15 Hz using nitrogen–oxygen gas mixture ^11,12^. This result suggested that U/VHFO alone may not be sufficient to promote effective CO_2_ excretion; however, administration of the helium–oxygen gas mixture would make U/VHFO effective in clinical settings.

More detailed analyses could have been conducted if the PaCO_2_ values before administering the helium–oxygen gas mixture had been within a narrower range. With the Humming Vue® ventilator used in this study, PaCO_2_ can be controlled by adjusting SV, frequency, or both. However, we prioritized in this study to minimize the variability of SV at each frequency as much as possible. Consequently, we accepted a broad range of PaCO_2_ values before the administration of the helium–oxygen gas mixture, which is the greatest limitation of our study.

The reasons for minimizing SV variability in this experiment are as follows. Initially, SV fluctuations might have complicated assessing the effects of the helium–oxygen gas mixture on PaCO_2_ during U/VHFO. Adjusting SV might alter tidal volume or alveolar ventilation when administering the helium–oxygen gas mixture. Such changes have been observed in experiments using HFOV (Sensomedics 3100B) ^21^, which operates on a different oscillation-generating mechanism compared to U/VHFO used in the present study. Therefore, minimizing the variability of SV was crucial to determine the combined effects of U/VHFO and helium–oxygen gas mixture. Moreover, an increase in SV undermines the basic principle underlying HFOV which uses very small oscillatory volumes (tidal volumes) that are smaller than the anatomical dead space and would not prevent lung injury ^16^. Considering future clinical applications, we determined that not significantly increasing SV was reasonable. Additionally, our previous study has demonstrated that adjusting SV to maintain PaCO_2_ within a narrow range is challenging, even at a constant frequency ^10^. Taking these factors and the survival of the experimental animals into account, we set the PaCO_2_ range before administering the helium–oxygen gas mixture to 70–95 mmHg.

Regarding PaO_2_, this study showed improvement with the administration of the helium–oxygen gas mixture and U/VHFO with 35 Hz and 45 Hz compared to that with 15 Hz. Oxygenation during HFOV was considered to depend on MAP and F_I_O_2_ separately, rather than SV or frequency. Therefore, theoretically, oxygenation should remain constant even with increasing ventilation frequency. Regarding the conflicting findings in our study, we postulate that the use of U/VHFO at higher frequencies may increase oscillatory pressure waves resulting in extreme elevation in airway resistance, resembling airway obstruction in the proximal airways. Administration of the helium–oxygen gas mixture may reduce the resistance of the proximal airways and relieve the slightly obstructed airways, and both ventilation and oxygenation could be maintained and recovered.

Combination therapy with HFOV and helium–oxygen gas mixture has been explored for some time ^20–24^. However, various types of HFOV instruments have also been used in the experiments and conflicting results have been reported, as different mechanisms of oscillation generation, that is, different shapes of pressure waveforms, were used as HFOVs ^32–34^. The U/VHFO used in this experiment was created by modifying the Humming Vue® software. When operating the Humming Vue® ventilator, both SV and frequency can be defined. A characteristic of the Humming Vue® is that, no matter what frequency, the piston moves in accordance with the set SV to generate oscillations. Metran Co., Ltd. confirmed that the prototype of the Humming Vue® operates according to the set SV even when working as a U/VHFO.

It is also known that the pressure waveform just above the endotracheal tube is sinusoidal irrespective of the frequency used (piston-driven, oscillations with sinusoidal waveform generated by piston) ^32–34^. Due to the configuration of the oscillation generator, the ratio of inspiratory-to-expiratory time is necessarily 1:1. The amplitude is measured at the tip of the ventilator circuit as a direct outcome of the ventilatory process. Some researchers interpret SV as a parameter analogous to tidal volume of Sensomedics 3100A/B, which will be discussed later.

Since the development of HFOV, many countries apart from Japan have utilized various HFOV devices that generate oscillations through different mechanisms. The Sensomedics 3100A/B is one such HFOV device. In the Sensomedics 3100A/B, oscillations are not directly generated by a piston but by the movement of a membrane when the amplitude/power (Δ pressure) is set. Although the inspiratory-to-expiratory time ratio is often defined as 1:2, other settings are possible. Thus, the pressure just above the endotracheal tube has a square waveform (piston-driven, oscillations with a square waveform generated by membrane)^32^. The major difference between the Sensomedics 3100A/B and Humming Vue® devices, in addition to their operating mechanisms, lies in the relationship between frequency and SV (tidal volume). In the Humming Vue® and other models of the Humming series, the same SV is obtained regardless of the frequency setting. Conversely, in the Sensomedics 3100 A/B, increasing the frequency results in a decrease in tidal volume^32^. Therefore, when using an HFOV that operates with a mechanism other than that driven by a piston-driven, piston generating oscillations with sinusoidal waveform as the U/VHFO, different results may be obtained.

One of the primary limitations of this study is that the pressure amplitude was not measured. As mentioned in the Methods section, pressure amplitude is not a pre-set parameter but rather a dependent variable in the Humming Vue® and was not specifically measured. The lack of accurate amplitude measurements raises the possibility that an increase in frequency was associated with an increase in pressure amplitude, contributing to CO_2_ excretion. Although the oscillation-generating mechanism of the Humming Vue® differs from that of the Sensomedics and other devices, if the SV is equivalent to the tidal volume of the Sensomedics 3100 A/B, increases in frequency might shorten the inspiratory time and increase the pressure amplitude measured at the tip of the ventilatory circuit. This amplitude increase may contribute to enhanced CO_2_ elimination. In contrast, the combination of U/VHFO with a helium–oxygen gas mixture may enable lower HFOV settings, such as reduced SV and/or F_I_O_2_, suggesting that lung-protective ventilation might be achievable. Additionally, while this study reported enhanced CO_2_ excretion and improved oxygenation, it did not examine whether HFOV settings could be relieved or whether lung protection could be achieved. Although the value of the amplitude is also displayed in the Humming Vue®, it was not specifically measured because the amplitude shown on the Humming Vue® display is accurate for measurements in the 5–17 Hz frequency range without a helium–oxygen gas mixture, which is the range of frequencies that can be set with the Humming Vue®. Amplitudes at higher frequencies generated by U/VHFO or amplitudes with a helium–oxygen gas mixture have not yet been verified. Analysing the displayed amplitude of the Humming Vue® might lead to incorrect conclusions.

Given that gas exchange in HFOV involves more alveolar diffusion than conventional ventilation ^11–15^, studying pressure amplitude and pressure fluctuations closer to the alveoli (e.g., at the distal end of the endotracheal tube, inside the trachea, or within the bronchi) is both biologically and physically important. We plan to measure these amplitudes using precise equipment in future experiments using U/VHFO.

Since HFOV aims to not only maintain oxygenation and ventilation but to also prevent ventilator-induced lung injury, future studies should focus on investigating the pathological and biochemical impacts of higher frequency settings in HFOV. These studies should specifically examine lung injury markers such as inflammatory cytokine levels, neutrophil infiltration, and alveolar integrity to better understand the potential lung-protective effects of HFOV combined with a helium–oxygen gas mixture.

The second limitation is our selection of 25, 35, and 45 Hz as the frequencies for U/VHFO, with each frequency differing by 10 Hz. The degree of pulmonary protection between 5 Hz HFOV and 15 Hz HFOV has been shown to be considerably different ^19^. It was shown that a difference of 10 Hz has a significant impact on the operation of HFOV. Previous studies that examined appropriate frequencies for HFOV adjusted frequencies by 2–3 Hz ^11^. In other words, there is a concern that changing the frequency by 10 Hz may be excessive. However, the purpose of this study was to examine the extent to which PaCO_2_ is altered by the administration of the helium–oxygen gas mixture in U/VHFO and to broadly determine which frequencies are promising for future treatment as U/VHFO. We consider that this purpose has been satisfactorily achieved. In the future, we plan to verify changes in PaCO_2_ when the frequency is changed using finer steps within the range of 30–40 Hz.

Future clinical applications include low-compliance pulmonary diseases such as acute respiratory distress syndrome (ARDS); however, these conditions require higher concentrations of inspiratory oxygen, so the concentration of inspiratory helium must be reduced. Therefore, some arguments that there are fewer opportunities to implement combined therapy of U/VHFO with helium–oxygen gas mixture can be anticipated. Nonetheless, this study has shown that 35 Hz U/VHFO improves not only ventilation but also oxygenation, and there may be an opportunity to utilise this combination therapy for ARDS in situations with progressive oxygenation deterioration. Furthermore, this combination therapy may be indicated in cases with relative contraindications to traditional HFOV, i.e., lesions with elevated airway resistance, such as bronchial asthma, air leak syndrome, and acute bronchiolitis. It has already been shown that helium–oxygen gas mixture decreases airway resistance in children suffering from acute bronchiolitis caused by respiratory syncytial virus ^25^. There have been reports of successful treatment with HFOVs in diseases with high airway resistance for which HFOV is conventionally indicated with caution ^35–37^. Therefore, it can be assumed that the combination therapy of HFOV with helium–oxygen gas mixture can be used to treat diseases with high airway resistance. Since this study showed that the combination therapy of U/VHFO with helium–oxygen gas mixture promotes CO_2_ excretion, it is expected to have the same effect in diseases with high airway resistance.

## Conclusion

This study showed that the combination therapy of a helium–oxygen gas mixture and high-frequency oscillation using ultra/very high frequencies (35–45 Hz) decreased PaCO_2_ and increased PaO_2_ more than that using a conventional frequency level (15 Hz) in a hypoventilated rabbit model with normal lungs. This combination therapy could normalise severe hypercapnia and hypoxemia in several diseases with impaired ventilation and hypoxia. The usefulness of this combination therapy in patients with lung pathology, such as decreased pulmonary compliance and/or increased airway resistance, requires further investigation. In addition, the lung-protective effect of this combination therapy needs to be further verified in histopathological, biochemical, and genetic studies.

## Data availability statement

The datasets analysed during the current study are available from the corresponding author on reasonable request.
